# Supplementary material for: COVID-19 related morbidity and mortality in people experiencing homelessness in the Netherlands
Source: PLoS One. 2024 Feb 5;19(2):e0296754. doi: 10.1371/journal.pone.0296754 (PMC10843097; doi:10.1371/journal.pone.0296754)
Supplement: Attachment — Submitted filename: Response to Reviewers.docx [file pone.0296754.s001.docx]

**Response to the reviewers**

**Reviewer #1:**

Dear reviewer, thank you for the valuable feedback. We have attempted to incorporate your suggestions into the manuscript as thoroughly as possible. Below, you will find point-by-point our response and modifications.

- Abstract conclusions and conclusions of the manuscript "Although SARS-COV-2 infection was not widespread among people experiencing homelessness in the Netherlands, the number of hospitalizations in this study was relatively high compared to the general population."
  this sentence is not supported by the study results because no "general population group" is provided as comparison.

We agree, we have changed both sentences and removed the part where we compare with general population.

- Please use "SARS-CoV-2" when referring to the infection and "COVID-19" when referring to the disease.

Changed accordingly

- Page 7 line 153-154 "2. Patients were categorized as clinically suspected if they were diagnosed with SARS-COV-2 but were not tested." please reword the sentence because it is not possible to diagnose SARS-CoV-2 infection without a molecular or antigenic test.

We have changed the sentence accordingly, emphasizing that diagnosis was not possible based on a molecular or antigenic test. We also added more information on how a diagnosis was made after a comment of reviewer #3.

*´Patients were categorized as clinically suspected if the street doctor diagnosed them with a SARS-CoV-2 infection, but confirmation through a molecular or antigenic test was not possible.”*

**Reviewer #3:**

Dear reviewer, thank you for the valuable feedback. We have attempted to incorporate your suggestions into the manuscript as thoroughly as possible. Below, you will find point-by-point our response and modifications.

- Since the Castor EDC form contained information from literature up to May 2020, this form was apparently designed after May 2020. I understand very well that such form could not yet be made at the onset of the epidemic. However, for clarity it would be useful that the authors would mention at what moment the Castor form was introduced - and to state that all data regarding patients seen before that moment had to be added in retrospect. This makes it even clearer that data at the onset of the epidemic were probably scanty and it is understandable that many data are missing - although I do not see a clue why "biological sex" was missing so often.

Thank you for this comment. We have added the information and agree that this is making it more clearer.

*“This form was created and finalized in May 2020 using an online survey programme Castor EDC. Items were based on literature available in May 2020 about COVID-19 and the expertise of experienced street doctors. For each patient the street doctor saw, a form was filled in with the required data. The data from March 2020 until May 2020 were added in retrospect. As of May 2020 , the street doctors received a reminder twice a month to enter the data of the patients they had seen in the previous two weeks.”*

A more fundamental comment that should have been raised regarding the first version of the manuscript is that there is no definition at all for "clinically suspected for SARS-CoV 2 infection". Therefore, the data as presented in table 3 regarding these groups will probably tell more about why the patients were put in this category. If clinicians, including street doctors, think that patients with fever are more likely to have SARS-CoV 2 infection, this will be reflected in these columns.
It is even less clear how patients were categorized as "clinically not suspected for SARS-CoV 2 infection". Apparently, this category includes both patients who presented with complaints after introduction of nationwide testing, but were not deemed eligible for testing by the clinician based on symptoms, and patients who presented with complaints before introduction of nationwide testing and might have been eligible for testing.
If I had reviewed the first version of the manuscript, I would have suggested to leave these categories -especially the last one- out of the manuscript. As it stands, it could also be sufficient to clarify these issues in the methods or in the result section.

We understand this comment very well and have again tried to improve the definition of the various categories (especially clinically suspected and clinically not suspected). We have chosen to keep the categories in the text. Especially because omitting them would give an even more biased presentation. In the Netherlands in the first part of the pandemic there was no testing policy, and certainly for homeless population there was no testing capacity available or in some cases people refused a test. Street doctors therefore had to base their diagnosis on multiple facts and observations from history and taking physical examination. In those cases where all symptoms indicate to COVID And doctors often could or did not perform a test, classified the case as "clinically suspect." And on the other hand, when fever or other complaints were more likely explained by other conditions, they will have classified this as "not-suspected". we have made adjustments to the text so that hopefully the definitions are more clear.

*´Patients were categorized as clinically suspected if the street doctor diagnosed them with a SARS-CoV-2 infection, but confirmation through a molecular or antigenic test was not possible. Particularly at the beginning of the pandemic, due to a shortage of PCR tests and policy guidelines, not all individuals experiencing homelessness who sought assistance from street doctors could be tested. Additionally, some individuals declined testing. Consequently, street doctors had to rely on multiple facts and observations from medical history and physical examinations to make a diagnosis. Cases in which all symptoms pointed to COVID-19, and a PCR test could not or was not performed, were classified as "clinically suspect." Conversely, when fever or other complaints were more likely attributed to other conditions, these cases were categorized as "not suspected" of a SARS-CoV-2 infection”*
